# The Mutation Patterns of *MET* Gene in Lung Cancer and Brain Tumors: Clinical and Therapeutic Implications

**DOI:** 10.1002/cam4.71532

**Published:** 2026-01-12

**Authors:** Yu Zhang, Ningning Luo, Minghui Ge, Yanxiang Zhang, Dongsheng Chen, Yongmeng Li

**Affiliations:** ^1^ Pulmonary and Critical Care Medicine The First Hospital of Hebei Medical University Shijiazhuang Hebei China; ^2^ State Key Laboratory of Neurology and Oncology Drug Development Jiangsu Simcere Diagnostics Co. Ltd., Nanjing Simcere Medical Laboratory Science Co. Ltd. Nanjing Jiangsu China; ^3^ Department of Thoracic Surgery The First Affiliated Hospital of Shandong First Medical University & Shandong Provincial Qianfoshan Hospital Jinan Shandong China

**Keywords:** brain tumors, clinical and therapeutic implications, different *MET* gene mutation types, lung cancer

## Abstract

**Objectives:**

*MET* aberrations are capable of triggering oncogenesis through multiple clinical significance genomic alterations. In non‐small cell lung cancer, *MET* exon 14 skipping and *MET* amplification confer sensitivity to MET tyrosine kinase inhibitors. The *MET* gene is also one of the druggable genes in high‐grade gliomas. However, a systematic comparison of *MET* variations between lung cancer and brain tumors is lacking.

**Methods:**

We analyzed a large Chinese cohort of 30,355 lung cancer and 6004 brain tumor patients. Different *MET* mutation types were characterized, and somatic genomic mutational characteristics were examined across various *MET* mutation subgroups in both lung cancer and brain tumor cohorts. The impact of *MET* mutations on prognosis in these two cohorts was also assessed. The study cohort underwent comprehensive genomic profiling using targeted next‐generation sequencing (NGS) panels.

**Results:**

We found that clinically significant *MET* mutations exist in both lung and brain tumor cohorts, with the lung cancer group having a higher overall frequency (*p* < 0.001), but the frequency of different *MET* mutation types, mutation characteristics, tumor mutation burden, and co‐mutated genes with high frequency all differ. *MET* alterations were significantly enriched in post‐treatment brain tumors (8.5% vs. 4.8% in treatment‐naïve, *p* < 0.001). *MET* mutations also have different prognostic effects in the two cancer types. *MET* alterations were not prognostic in lung cancer but were associated with significantly poorer survival in brain tumors (median OS: 19.9 vs. 62.9 months, *p* < 0.001), a finding that held in multivariate analysis.

**Conclusions:**

Our study demonstrated that the biological and clinical significance of *MET* alterations is highly context dependent. In lung cancer, *MET* serves primarily as a predictive biomarker for targeted therapy, whereas in brain tumors, it functions as a prognostic marker of genomic instability and aggressive disease. These findings advocate for context‐specific clinical management strategies.

## 1 Introduction

The *MET* gene, also known as c‐Met or HGFR (Hepatocyte Growth Factor Receptor), encodes a transmembrane receptor tyrosine kinase (RTK), which has attracted considerable attention in oncology due to its significant role in tumorigenesis and metastasis [1]. *MET* gene aberrations are recognized as a key driver of oncogenesis, playing a prominent role in the development and progression of various malignancies [2, 3, 4]. The *MET* gene is distinguished from other proto‐oncogenes by its capacity to trigger oncogenesis through three genomic alterations with clinical significance: amplification, mutation, and fusion. *MET* mutations include those resulting in exon 14 skipping and those affecting the kinase or extracellular domains [5]. There is growing evidence that targeted therapies can be beneficial for patients with tumors exhibiting *MET* alterations [5, 6, 7].

In oncogene‐driven non‐small‐cell lung cancer (NSCLC), targeted therapies have revolutionized treatment and significantly improved patient outcomes, with an expanding number of oncogenic driver therapies becoming available [8]. NSCLC may harbor various *MET* alterations [9]. Splice site mutations in *MET* have been found to cause exon 14 skipping, which increases MET protein levels by disrupting the receptor's normal ubiquitin‐mediated degradation process [10, 11]. Approximately 2%–4% of advanced NSCLC cases feature *MET* exon 14 skipping mutations or deletions [9, 10, 11, 12, 13]. Patients with NSCLC harboring *MET* exon 14 skipping mutations are sensitive to MET tyrosine kinase inhibitors (TKIs), and a variety of MET TKIs are available [8, 14, 15]. *MET* copy number gain, present in about 1%–6% of NSCLC patients, occurs via polysomy or regional/focal amplification. Polysomy refers to the presence of multiple copies of chromosome 7 carrying the *MET* gene, while amplification indicates an increase in the copy number of the *MET* gene itself, leading to protein overexpression. It is crucial not to distinguish true gene amplification, which results from gene duplication, from an increase in *MET* copy number due to high polysomy [8, 9, 16]. Multiple studies have indicated that *MET*‐amplified advanced NSCLC patients exhibit an objective response rate (ORR) of 30%–40%, with higher amplification levels associated with a greater probability of responding to MET TKI therapy [8, 16, 17].

Primary malignant brain and other central nervous system (CNS) tumors are among the most lethal cancers, characterized by a high mortality rate, and the most common malignant brain tumor is glioma [18]. Activating mutations in the *MET* gene are pivotal in the transition of low‐grade gliomas to secondary glioblastomas and contribute to the pathogenesis of glioblastoma [19]. Additionally, increased MET expressions in diffuse astrocytomas are linked to a poor prognosis [20]. The *MET* gene is one of the druggable genes in high‐grade gliomas [21, 22]. Research has revealed the presence of *MET* fusion transcripts in secondary glioblastomas, highlighting their potential as a drug target in both adult and pediatric glioblastoma cases [19, 23, 24]. The role of *MET* exon 14 in secondary glioblastomas has been clarified, showing its contribution to glioma progression. Furthermore, the co‐occurrence of *MET* exon 14 and other fusions is linked to an increase in tumor‐associated macrophages, a factor associated with a poor prognosis in glioma patients [19].

Activating oncogenic mutations in *MET* can also occur in various domains, including the tyrosine kinase domain (TKD), resulting in ligand‐independent receptor phosphorylation and signaling. Moreover, mutations affecting the Sema domain in the extracellular region have also been identified in cancer [5, 7]. Next‐Generation Sequencing (NGS) can simultaneously detect *MET* fusions, amplifications, exon 14 skipping, and other mutations affecting the kinase or extracellular domains. With the increased application of tumor‐ and blood‐based NGS, these alterations are being identified more frequently in patients whose tumors undergo broad panel‐based sequencing analysis [25]. However, there has been no systematic comparative study of *MET* variations in lung cancer and brain tumors, and it is worth exploring whether the relatively mature targeted therapy model for lung cancer can offer insights for brain tumors. In our study, we analyzed the spectrum of different *MET* mutation types in both the lung cancer and brain tumor cohorts and further examined the somatic genomic mutational characteristics across various *MET* mutation subgroups. Additionally, we utilized data from The Cancer Genome Atlas (TCGA) to analyze the impact of *MET* mutations on prognosis in these two cohorts. This research provides a better understanding of *MET* mutation characteristics in lung and brain tumors, offering further insights into potential treatments for patients with different *MET* mutations in these cancers.

## 2 Materials and Methods

### 2.1 Patient Cohort and Sample Collection

A total of 30,355 patients with lung cancer and 6004 patients with brain cancer diagnosed between June 2019 and October 2024 from the Simceredx cohort were enrolled in this study. We collected baseline characteristics of all participants, including age, gender, and cancer type. Comprehensive genomic profiling was performed on the study cohort using targeted next‐generation sequencing panels (a 551‐gene tumor panel, the Neuro‐onco360 panel, or a 131‐gene panel) by Simcere Diagnostics Co. Ltd. in Nanjing, China. This study was conducted in accordance with the principles of the Declaration of Helsinki. For this retrospective analysis, the requirement for written informed consent was waived.

### 2.2 Next‐Generation Sequencing Detection

Genomic DNA (gDNA) was extracted by two kits: a gDNA Tissue Extraction Kit (Concert) was employed to extract gDNA from formalin‐fixed paraffin‐embedded tumor samples or fresh frozen tumor tissues, while paired leukocyte gDNA was prepared using a magnetic universal gDNA kit (TIANGEN). The Qubit dsDNA HS Assay Kit quantified the extracted gDNA with a Thermo Fisher Scientific Qubit Fluorometer, and the quality of the gDNA was assessed on an Agilent 4200 TapeStation.

Subsequently, 200 ng of gDNA was sheared into 200–300 bp fragments via enzymatic fragmentation. The KAPA Hyper DNA Library Preparation Kit from Roche Diagnostics was used for end‐repair of the sheared DNA. The VAHTSTM Universal DNA Library Prep Kit for Illumina (Vazyme) was then used for A‐tailing and the ligation of indexed paired‐end adaptors, compatible with the SimcereDx Illumina platform. Agencourt AMPure XP beads were employed for size selection to eliminate unligated adaptors. The ligation products were amplified via PCR to create a hybridization pre‐library.

The final qualified DNA libraries were sequenced using 150‐bp paired‐end sequencing on the Illumina NovaSeq6000 platform, following the manufacturer's guidelines, at the CAP and CLIA‐accredited central laboratory of Jiangsu Simcere Diagnostics Co. Ltd. in Nanjing, China.

### 2.3 Bioinformatics Analysis

Raw sequencing data were converted to FASTQ format, and quality control was performed using fastp software (V.2.20.0) to trim adapters and remove low‐quality bases [26]. The cleaned paired‐end reads were aligned to the hg19 human genome reference (UCSC hg19/GRCh37) using the Burrows‐Wheeler Aligner (BWA‐MEM v.0.7.17) [27]. PCR duplicates were removed with Dedup. Single nucleotide variants (SNVs) and small insertions/deletions (InDels) were identified and annotated using VarDict (v.1.5.7) and InterVar. The identified variants were screened against public databases for common SNPs, including the 1000 Genomes Project (Aug 2015) and ExAC Browser (v.0.3). Fusions and copy number variations (CNVs) were assessed using Factera (v1.4.4) and CNVkit (v1.1), respectively. Tumor mutation burden (TMB) was calculated as the total number of nonsynonymous somatic mutations in the coding region per megabase, excluding known somatic alterations listed in the COSMIC database.

### 2.4 *MET* Variant Classification

*MET* alterations were classified into several major categories: (1) *MET* exon 14 skipping, encompassing mutations at the splice‐acceptor or ‐donor sites of introns 13 and 14, mutations within exon 14 itself, and specific missense mutations (Y1003F/N/S and D1010H/N/Y) known to cause exon 14 skipping; (2) *MET* fusions, defined as all fusion events involving the *MET* kinase domain (exons 15–21); (3) *MET* amplification, categorized based on the multiples of copy number into *MET* CNV 2–5, *MET* CNV 5–10, and *MET* CNV ≥ 10. This category also includes segmental amplifications, defined as copy number gains encompassing the *MET* locus and its adjacent genomic regions; (4) mutations in the MET kinase domain; (5) mutations in the MET Sema domain. Cases harboring more than one type of the above *MET* alterations were defined as *MET* Multi, and cases containing any of the five categories listed above were defined as *MET* variant (*MET* var).

### 2.5 Data Acquisition and Analysis From TCGA

We obtained clinical and genomic data for brain and lung cancer from The Cancer Genome Atlas (TCGA). The lung cancer cohort comprised 1075 cases, including 507 cases of lung adenocarcinoma (LUAD) and 495 cases of lung squamous cell carcinoma (LUSC). The brain cancer cohort included 1105 cases, consisting of 509 cases of low‐grade glioma (LGG) and 581 cases of glioblastoma multiforme (GBM). All data were downloaded from the UCSC Xena platform (https://xena.ucsc.edu/). Detailed methodologies for CNV pipelines and gene fusion detection pipelines are available on the Genomic Data Commons (GDC) documentation website.

### 2.6 Statistical Analysis

All statistical analyses were performed using R (version 4.4.1). The somatic mutation landscape was visualized using the oncoplot function from the maftools package (version 2.18.0), while lollipop plots illustrating amino acid changes in *MET* were generated using the lollipopPlot function. Group comparisons for different *MET* mutation types were conducted using the Wilcoxon rank‐sum test, while the *t*‐test was applied for evaluating age‐related differences. Survival analysis was carried out with the survminer (version 0.4.9) and survival (version 0.4.9) packages, with Kaplan–Meier survival curves plotted using the ggsurvplot function and differences assessed through the log‐rank test. These methods ensured rigorous and comprehensive statistical evaluation. A two‐sided *p* value < 0.05 was considered to indicate statistical significance.

## 3 Results

### 3.1 Patient Enrollment and the Detection of *MET* Variation in Lung Cancer and Brain Tumor Cohorts

A total of 30,355 patients were enrolled in the lung cancer cohort, of which 3200 cases tested positive for any *MET* alterations. These included 364 cases of *MET* exon 14 skipping, 15 of *MET* fusions, 2159 of *MET* CNV 2–5, 188 of *MET* CNV 5–10, 60 of *MET* CNV ≥ 10, 153 of MET kinase domain mutations, 398 of MET Sema domain mutations, and 131 cases with multiple *MET* alterations. The brain tumor cohort enrolled a total of 6004 patients, with 331 cases testing positive for any *MET* alterations. These included 5 cases of *MET* exon 14 skipping, 12 of *MET* fusions, 189 of *MET* CNV 2–5, 35 of *MET* CNV 5–10, 47 of *MET* CNV ≥ 10, 23 of MET kinase domain mutations, 44 of MET Sema domain mutations, and 23 cases with multiple *MET* alterations. The number of patients in each group is detailed in Table S1.

**FIGURE 1:**
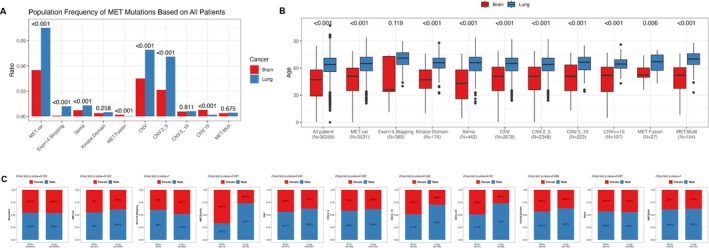
The detection rates of *MET* variation and clinical characteristics of different *MET* alteration types in lung cancer and brain tumor cohorts. (A) The detection rates of different *MET* alteration types. The age (B) and gender (C) in different *MET* alteration types across the two cohorts.

The detection rates of different *MET* alteration types in the two cancer cohorts are shown in Figure 1A. There was a statistically significant difference in the overall *MET* alteration frequency between the lung cancer and brain tumor groups, with the lung cancer group having a higher rate (*p* < 0.001). Fusions and CNV ≥ 10 are more frequent in the brain tumor group (*p* < 0.001), while exon 14 skipping, Sema domain mutations, overall CNV, and CNV 2–5 are more frequent in the lung cancer group (*p* < 0.001). There was no significant difference in the frequency of kinase domain mutations, CNV 5–10, or *MET* multi between the two cohorts.

Statistical analysis of age (Figure 1B) across the two cohorts revealed that there are statistically significant differences in age between the lung cancer and brain tumor cohorts and across different *MET* mutation types except for exon 14 skipping, with the lung cancer group being older. In terms of gender (Figure 1C), there was no overall statistical difference between the lung cancer and brain tumor cohorts. However, when focusing on CNV, especially high‐level CNV (CNV 5–10 and CNV ≥ 10), a statistically significant difference was observed between the two cohorts, with a higher proportion of females in the brain tumor group (*p* < 0.05) than in the lung cancer group. No other subgroups showed statistical significance in terms of gender distribution.

Treatment history was available in the brain tumor cohort. Analysis of this subgroup revealed a significantly higher frequency of *MET* alterations in recurrence/treated samples (8.5%, 40/470) compared to primary samples (4.8%, 252/5242) (*p* < 0.001, Chi‐square test) (Figure S1).

### 3.2 The Distinct Types of *MET* Variations in the Lung Cancer Cohort and Brain Tumor Cohort

**FIGURE 2:**
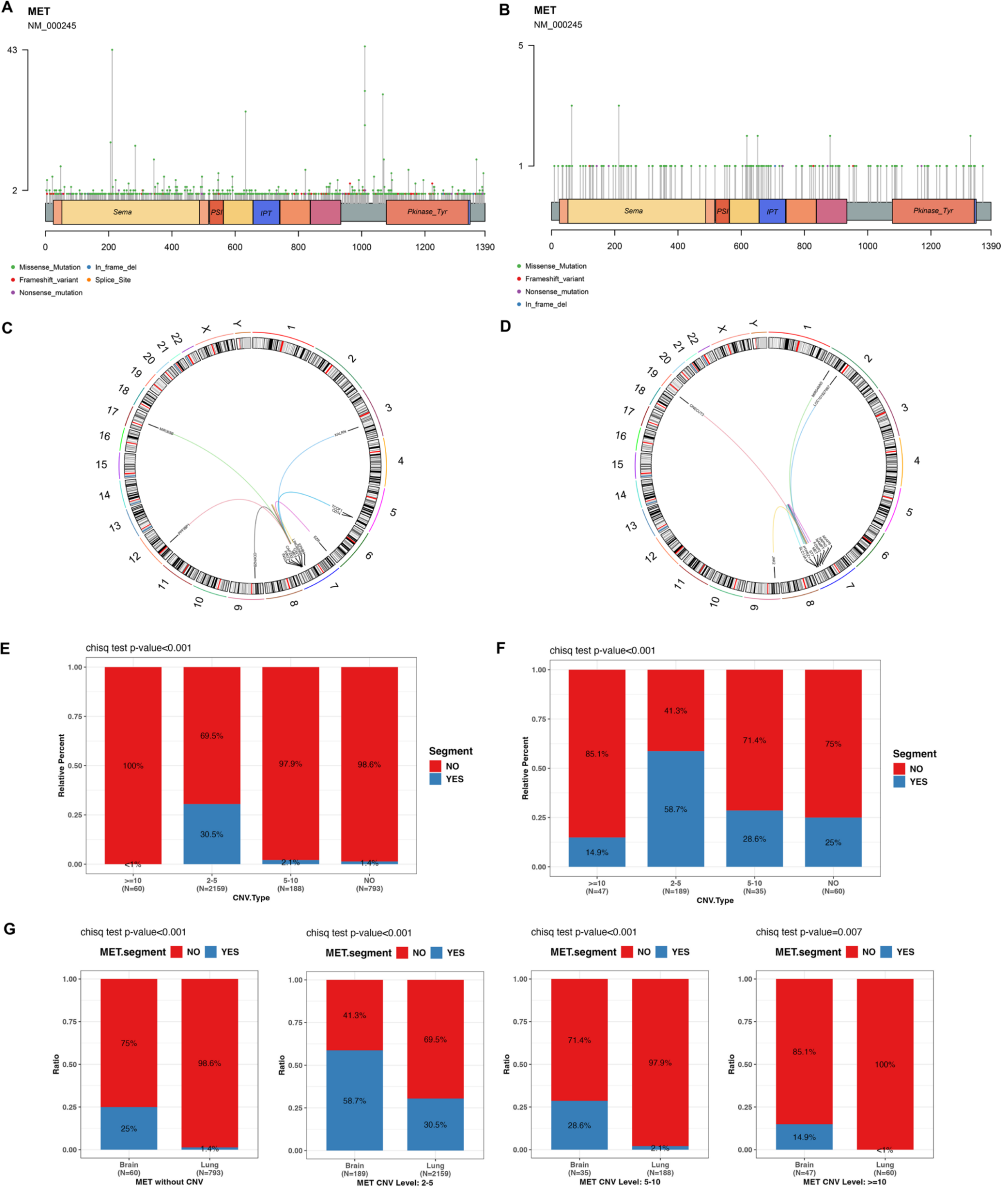
The distinct types of *MET* variations in the lung cancer and brain tumor cohorts. The lollipop plot of all *MET* gene SNVs and Indel mutations across the lung cancer cohort (A) and the brain tumor cohort (B). The Circos plots depicting *MET* fusions in lung cancer cohort (C) and brain tumor cohort (D). The co‐occurrence of *MET* CNV and Segmental CNVs in different CNV subgroups in lung cancer cohort (E) and brain tumor cohort (F). Comparative analysis of co‐occurrence of Segmental CNVs across two cohorts (G).

Lollipop plot illustrates the locations and protein domains of all *MET* gene SNVs and Indel mutations across the lung cancer cohort (Figure 2A) and the brain tumor cohort (Figure 2B). In the lung cancer cohort, *MET* mutations exhibited high frequency of hotspots in front of the Kinase domain and within the Sema domain. In contrast, no significant hotspot mutations were observed in other regions or in the brain tumor cohort.

Circos plots depict *MET* fusions in the lung cancer (Figure 2C) and brain tumor (Figure 2D) cohorts. In the brain tumor cohort, *MET* fusion partner genes were predominantly located on chromosome 7 (9 out of 12 cases), with only 3 inter‐chromosomal fusions involving chromosomes 2, 9, and 19. Similarly, in the lung cancer cohort, *MET* fusion partner genes were also enriched on chromosome 7 (10 out of 15 cases), and the remaining five were inter‐chromosomal fusions involving chromosomes 3, 5, 6, 9, 12, and 17.

We analyzed the co‐occurrence of *MET* CNV and Segmental CNVs in different CNV subgroups in both cohorts (Table 1) and found that both brain and lung cohorts had a higher proportion of co‐occurrence of Segmental CNVs in the CNV 2–5 group (*p* < 0.001) (Figure 2E,F). Comparative analysis between the cohorts revealed that the brain cohort had a higher proportion of co‐occurrence of Segmental CNVs, suggesting a relatively higher degree of genomic instability (*p* < 0.01) (Figure 2G).

**TABLE 1:** The co‐occurrence of *MET* CNV and Segmental CNV in different CNV subgroups within the two cohorts.

	CNV	Segment	No segment	Total
Brain cancer	≥ 10	7	40	47
	5–10	10	25	35
	2–5	111	78	189
Lung cancer	≥ 10	0	60	60
	5–10	4	184	188
	2–5	659	1500	2159

### 3.3 Genomic Mutational Characteristics of Different *MET* Mutation Groups in Lung Cancer and Brain Tumor Cohorts

#### 3.3.1 Somatic Mutations and Mutational Signatures

**FIGURE 3:**
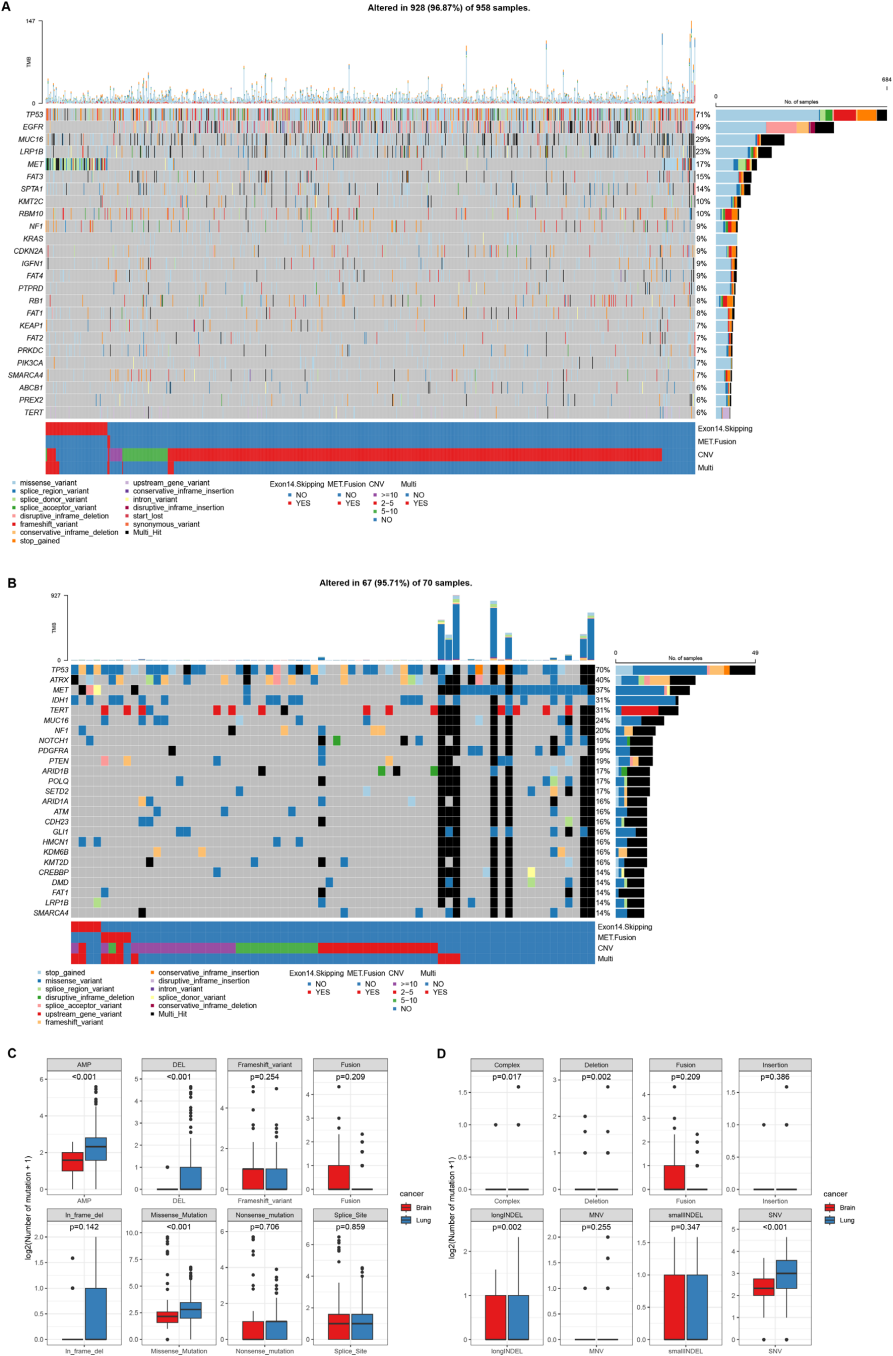
The somatic mutations and mutational signatures of different *MET* mutation groups in lung cancer and brain tumor cohorts. The oncoprint plot for the top 25 mutated genes among lung cancer cohort (A) and brain tumor cohort (B) with *MET* mutations. The comparison of the somatic mutational characteristics between the two cohorts with *MET* mutations (C and D).

We generated an oncoprint of the top 25 mutated genes among lung cancer cohort with *MET* mutations (Figure 3A). Besides *MET*, the top three most frequently mutated genes were *TP53* (allele frequency, AF, 71%), *EGFR* (AF, 49%), and *MUC16* (AF, 29%). Missense mutations were the most common variant type. Notably, we found no co‐occurrence of *KRAS* gene mutations in cases with *MET* exon 14 skipping or *MET* fusions, and co‐occurrence with *EGFR* gene mutations was also rare, observed in only two cases. For the brain tumor cohort, an oncoprint plot of the top 25 mutated genes was created for patients with *MET* mutations (Figure 3B). Aside from *MET*, the top three mutated genes were *TP53* (AF, 70%), *ATRX* (AF, 40%), and *IDH1* (AF, 31%). In addition to missense mutations, a higher proportion of multiple mutation types was observed. Patients with multiple *MET* alterations (*MET* Multi) tended to harbor a greater number of concurrent mutations.

Next, we compared the somatic mutational characteristics between the two cohorts with *MET* mutations (Figure 3C,D). We found that the average number of mutations in Copy number gain (Amplification, AMP, *p* < 0.001), Copy number loss (Deletion, DEL, *p* < 0.001), Missense_Mutation (*p* < 0.001), Complex (*p* < 0.05), Deletion (*p* < 0.01), longIndel (*p* < 0.01), and SNV (*p* < 0.001) were significantly higher in the lung cancer cohort compared to the brain tumor cohort. Additionally, we analyzed the differences in somatic mutational characteristics between groups with and without *MET* alterations within each cohort. In the lung cancer cohort (Figure S2A,B), the *MET* var. group had a significantly higher average mutation frequency in AMP (*p* < 0.001), DEL (*p* < 0.001), Missense_Mutation (*p* < 0.001), Nonsense_Mutation (*p* < 0.01), Splice_Site (*p* < 0.001), and SNV (*p* < 0.001) mutations compared to the Non‐*MET* group. Similarly, in the brain tumor cohort (Figure S2C,D), the *MET* mutation group exhibited a significantly higher average number of mutations in AMP (*p* < 0.001), Missense_Mutation (*p* < 0.001), Nonsense_Mutation (*p* < 0.001), Splice_Site (*p* < 0.05), Complex (*p* < 0.001), and SNV (*p* < 0.001) mutations compared to the Non‐*MET* group.

#### 3.3.2 Analysis of TMB and Segmental CNVs in the Two Cohorts

**FIGURE 4:**
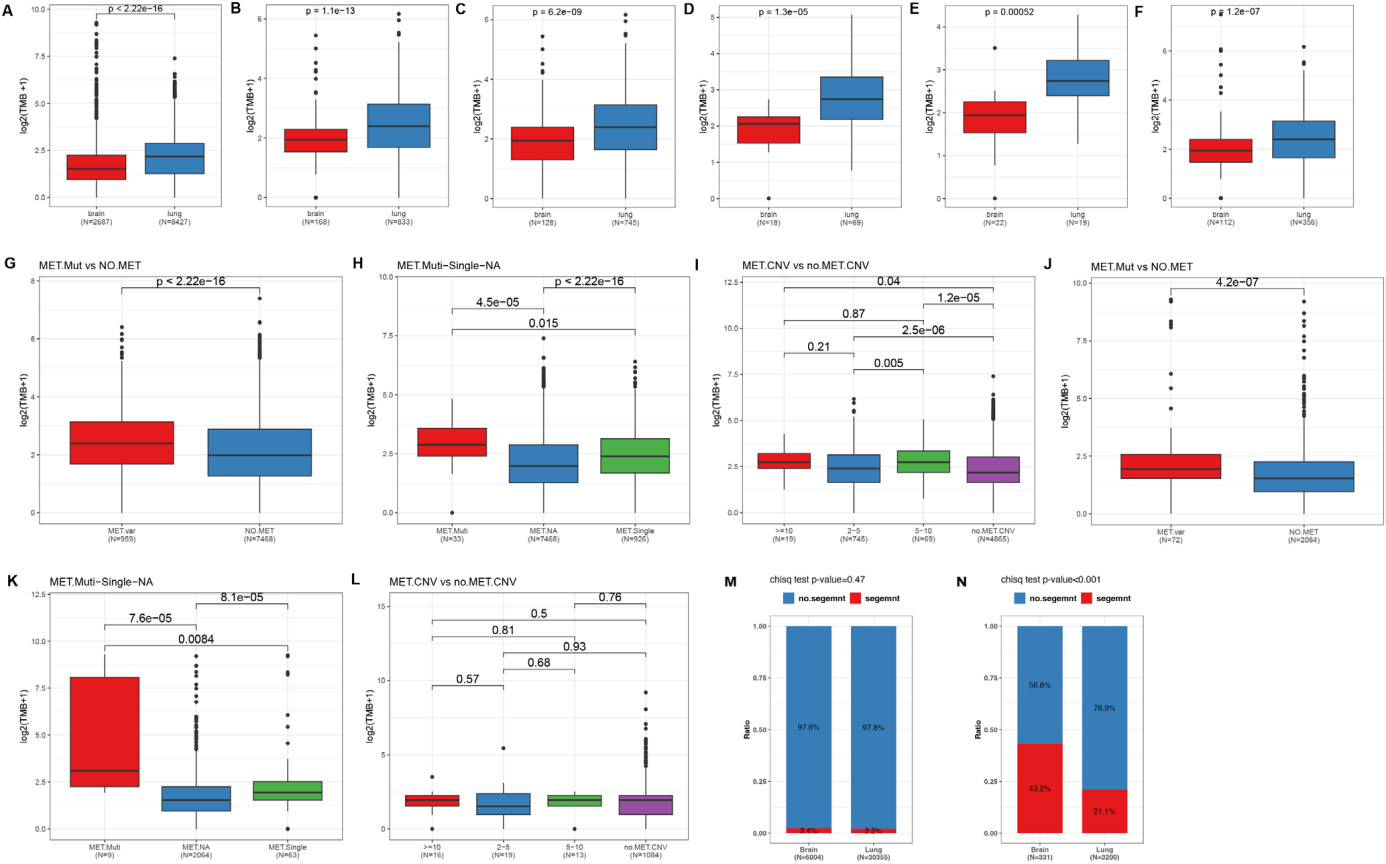
The differences in tumor mutational burden (TMB) and segmental CNVs in different groups. The difference on TMB in overall populations (A), in CNV (B), CNV2‐5 (C), CNV 5–10 (D), CNV ≥ 10 (E), and *MET* Segment (F) groups of the two cohorts. The TMB differences among *MET* mutation group and non‐*MET* group (G), *MET* single mutation, *MET* multi‐mutation, and non‐*MET* groups (H), different *MET* CNV groups (I), within the lung cancer cohort. The comparison of TMB differences among *MET* mutation group and non‐*MET* group (J), *MET* single mutation, *MET* multi‐mutation, and non‐*MET* groups (K), different *MET* CNV groups (L), within the brain tumor cohort. The differences in the proportion of segmental CNVs in the two cohorts of total population (M) and in the *MET* mutation groups across the two cohorts (N). TMB values are displayed on a log2 scale.

We compared the differences in TMB between the lung cancer and brain tumor cohorts, both overall and within different *MET* mutation subgroups (Figure 4 and Figure S3). The TMB was significantly higher in the lung cancer cohort than in the brain tumor cohort across the overall populations (Figure 4A) and within the following subgroups: all *MET* CNV (Figure 4B), CNV2‐5 (Figure 4C), CNV 5–10 (Figure 4D), CNV ≥ 10 (Figure 4E) and *MET* segmental amplification (Figure 4F) (*p* < 0.001 for all; Wilcoxon test). Conversely, within the *MET* Kinase Domain mutation subgroup, the brain tumor cohort exhibited a significantly higher TMB than the lung cancer cohort (*p* = 0.016; Figure S3A). No statistically significant differences in TMB were observed for the other *MET* subgroups (*p* > 0.05; Wilcox test) (Figure S3B–E).

We next compared TMB among different *MET* mutation subgroups within each cohort. In the lung cancer cohort (Figure 4G), the *MET* mutation group had a significantly higher TMB than the non‐*MET* group (median TMB 4.26 vs. 2.94, *p* < 0.001). Further subdividing into *MET* single mutation, *MET* multi mutation, and non‐*MET* groups revealed that both the *MET* single group (*p* < 0.001) and the *MET* multi group (*p* < 0.001) had higher TMBs than the non‐*MET* group, with the *MET* multi group exhibiting an even higher TMB compared to the *MET* single group (median TMB 6.38 vs. 4.26, *p* < 0.05) (Figure 4H). Compared to the non‐*MET* CNV group (median TMB 3.55), the CNV2‐5 group (median TMB 4.26, *p* < 0.001), CNV 5–10 group (median TMB 5.67, *p* < 0.001), and CNV ≥ 10 group (median TMB 5.67, *p* < 0.05) all had significantly higher TMBs (Figure 4I).

When comparing TMB differences among different *MET* mutation subgroups within the brain tumor cohort, we found the similar results: the *MET* mutation group had a higher TMB compared to the non‐*MET* group (median TMB 2.83 vs. 1.89, *p* < 0.001) (Figure 4J); both the *MET* single mutation group (*p* < 0.001) and the *MET* multi mutations group (*p* < 0.001) had higher TMBs than the non‐*MET* group, with the *MET* multi mutations group having a higher TMB compared to the *MET* single mutation group (median TMB 7.55 vs. 2.83, *p* < 0.01) (Figure 4K). However, unlike the lung cancer cohort, no statistical differences were observed among the various CNV subgroups (Figure 4L).

Finally, we analyzed the proportion of segmental CNVs in the two cohorts and found no statistical difference in the overall proportion of harboring segmental CNVs (2.4% vs. 2.2%, *p* = 0.47, Figure 4M). However, there was a statistically significant difference in the proportion of segmental CNVs among samples with *MET* mutations, with a higher proportion in the brain cohort (43.2% vs. 21.1%, *p* < 0.001, Figure 4N).

#### 3.3.3 CNV Burden and Chromosome Arm Alterations in Different *MET* Mutation Groups in Brain Tumors

**FIGURE 5:**
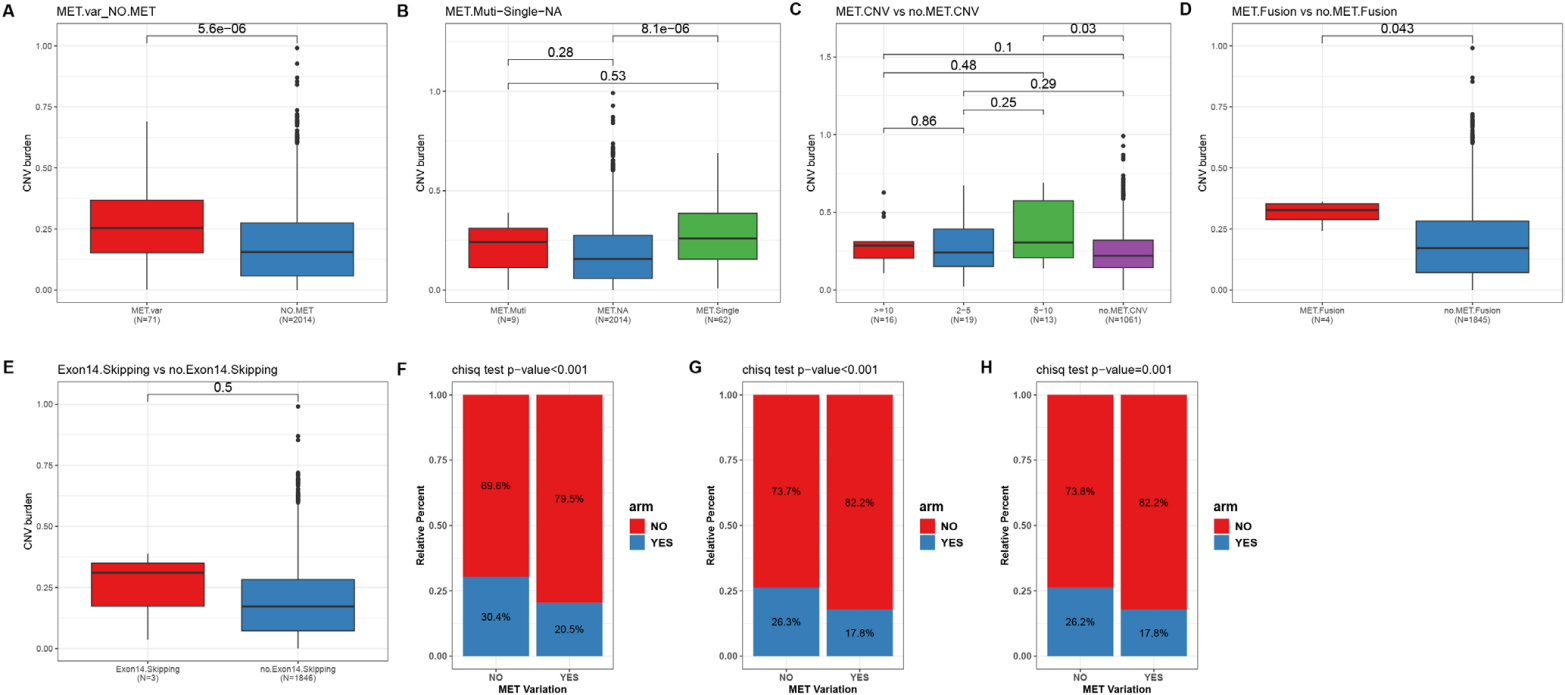
CNV burden and chromosome arm alterations in different *MET* mutation groups in brain tumors. CNV burden analysis in *MET* var. group and non‐*MET* group (A), in *MET* single‐mutation group, *MET* multi‐mutations group and non‐*MET* group (B), in different CNV subgroup (C), in *MET* fusion and no *MET* fusion groups (D), in *MET* exon 14 skipping and no exon 14 skipping groups (E). The chromosome arm copy number alterations analysis of overall proportion of arm‐level CNVs (F), arm‐level gains (G) and losses (H) in the *MET* var. and non‐*MET* groups.

We further analyzed the CNV burden and copy number alterations of chromosome arms (ARM) within the brain tumor cohort. The *MET* var. group exhibited a significantly higher CNV burden than the non‐*MET* group (median CNV burden: 0.2538 vs. 0.15575, *p* < 0.001; Figure 5A). Specifically, the *MET* single mutation group (median CNV burden: 0.2589) showed a significantly elevated CNV burden compared to the non‐*MET* group (*p* < 0.001), whereas the *MET* multi mutations group (median CNV burden: 0.241) also had a higher CNV burden, although this difference was not statistically significant (Figure 5B). The *MET* CNV 5–10 group exhibited a higher CNV burden compared to the non‐CNV group (median CNV burden: 0.3049 vs. 0.2206, *p* < 0.05) (Figure 5C). Additionally, cases with *MET* fusions demonstrated a higher CNV burden than those without fusions (median CNV burden: 0.3271 vs. 0.1716; *p* < 0.05; Figure 5D). In contrast, no significant difference in CNV burden was observed between subgroups with and without *MET* exon 14 skipping (Figure 5E). We also assessed chromosome arm‐level copy number alterations (Figure S4). Compared to the *MET* var. group, the non‐*MET* mutation group had a significantly higher overall proportion of arm‐level CNVs (30.4% vs. 20.5%, *p* < 0.001, Figure 5F), as well as arm‐level gains (26.3% vs. 17.8%, *p* < 0.001, Figure 5G) and losses (26.3% vs. 17.8%, *p* < 0.001, Figure 5H).

### 3.4 Impact of Different *MET* Mutations on Prognosis in the Two Cohorts

**FIGURE 6:**
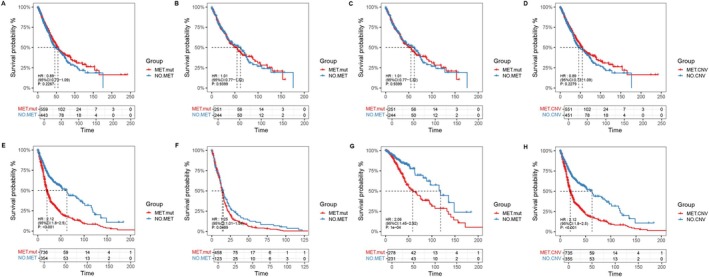
Impact of different *MET* mutations on prognosis in the two cohorts. The prognosis analysis between the *MET* var. group and the non‐*MET* group in overall lung cancer (A), in LUAD (B) and LUSC (C). The prognosis analysis between the *MET* CNV and the non‐*MET* CNV in overall lung cancer (D). The prognosis analysis between the *MET* var. group and the non‐*MET* group in overall brain tumor (E), in GBM (F) and LGG (G). The prognosis analysis between the MET CNV and the non‐*MET* CNV in overall brain tumor (H).

To explore the prognostic impact of *MET* mutations, we conducted an analysis using mutation and clinical outcome data from The Cancer Genome Atlas (TCGA). The number of patients in each group within TCGA is detailed in Table S1. In the lung cancer cohort, we found no significant differences in overall survival (OS) between the *MET* var. group and the non‐*MET* group, both in the overall population (Figure 6A) and in the LUAD (Figure 6B) and LUSC (Figure 6C) subgroups. Similarly, no significant survival differences were observed among the various *MET* mutation subgroups (Figure 6D and Figure S5).

In contrast, within the brain tumor cohort, the *MET* var. group exhibited significantly poorer OS compared to the non‐*MET* group (median OS: 19.9 vs. 62.9 months, *p* < 0.001, Figure 6E). Further subgroup analysis showed similar results in GBM (median OS: 14.1 vs. 15.6 months, *p* < 0.05, Figure 6F) and LGG (median OS: 58.7 vs. 119 months, *p* < 0.001, Figure 6G). Similarly, the *MET* CNV group also had significantly poorer survival compared to the no‐*MET* CNV group (median OS: 19.9 vs. 62.9 months, *p* < 0.001, Figure 6H). No significant survival differences were found among other *MET* mutation subgroups (Figure S5).

To account for potential confounders, a multivariate Cox regression analysis was performed, adjusting for age, gender, IDH status, and tumor grade. The analysis confirmed that *MET* variation remained an independent predictor of poor overall survival in the brain tumor cohort (Hazard Ratio [HR] = 1.28, 95% Confidence Interval [CI]: 1.047–1.562, *p* = 0.0158) (Figure S6).

## 4 Discussion and Conclusion

*MET* variations are rare in clinical practice, but MET is well known as a therapeutic target for lung cancer. MET inhibitors have shown good efficacy in clinical trials [17, 19, 28, 29, 30]. However, the complexity and heterogeneity of drug resistance mechanisms are still one of the difficulties in current research. Therefore, this study systematically classified *MET* variations and compared their differences in mechanisms of roles in lung cancer and brain cancer.

*MET* alterations occur in both lung cancer and brain tumors. Our comparative analysis reveals fundamentally distinct biological and clinical implications for *MET* alterations in lung cancer versus brain tumors, supporting the concept of different *MET* ecologies. In lung cancer, *MET* alterations, particularly exon 14 skipping, often function as primary oncogenic drivers. This is evidenced by their mutual exclusivity with other potent drivers, such as the absence of *KRAS* co‐mutations in exon 14 skipping or fusion cases and rare co‐occurrence with *EGFR*. This genetic pattern is characteristic of a single dominant driver scenario, underscoring the high predictive value of *MET* alterations for response to MET TKIs in this setting [8, 14, 15]. In stark contrast, brain tumors were more frequent in *MET* fusions, high‐level copy gains (CNV ≥ 10), and higher burden of segmental CNVs, a hallmark of genomic instability. This, coupled with a significant enrichment of *MET* alterations in recurrence/treated samples, positions *MET* in brain tumors not as a founding driver but as a progression event. Consequently, its strong association with poorer overall survival, independent of established prognostic factors, establishes *MET* as a powerful prognostic biomarker in this context.

This fundamental biological difference explains the divergent prognostic impact observed. In the lung cancer cohort, where *MET* is a therapeutically actionable driver, the widespread use of MET TKIs and the flatting of prognostic effects in effectively treated driver‐defined populations likely account for the lack of overall survival difference [8, 17]. Conversely, in brain tumors, *MET* events emerge later, often at recurrence, correlating with focal high‐level amplification, higher CNV burden, and significantly poorer OS, independent of age, IDH status, and tumor grade in multivariate analysis (HR = 1.28, *p* = 0.016). The significant enrichment of *MET* alterations in recurrent brain tumors underscores its role as a potent biomarker of disease progression. These findings support routine *MET* testing in gliomas, expanded access to MET inhibitors in fusion‐positive disease, and prospective trials evaluating combination therapy and resistance mechanisms, particularly in tumors with multiple *MET* alterations. In addition, our data argue strongly for the clinical utility of serial molecular profiling in brain tumor management. Re‐biopsy and genomic re‐evaluation at the time of progression are crucial to identify emergent, therapeutically actionable *MET* amplifications or fusions that were absent at initial diagnosis, thereby guiding subsequent lines of targeted therapy.

*MET* CNV ≥ 10 is enriched in brain tumor cohort and *MET* CNV 5–10 group exhibited a higher CNV burden compared to the non‐CNV group, confirming its link to a more unstable genomic phenotype. Most importantly, patients in the *MET* CNV group had significantly poorer overall survival compared to those without *MET* CNV (median OS 19.9 vs. 62.9 months, *p* < 0.001). These results indicate that the *MET* amplification subgroup is clinically actionable as a risk stratifier and glioma patients with *MET* amplification represent a biologically distinct, high‐risk subgroup. A higher overall CNV burden in *MET*‐altered cases yet a greater prevalence of arm‐level events in the non‐*MET* group suggests that the genomic instability in *MET*‐variant brain tumors is distinct. It is consistent with a model of focal, oncogene specific amplification centered on the *MET* locus (7q31), rather than being a consequence of broad, chromosomal‐scale aneuploidy. An intriguing finding was the higher proportion of females in the brain tumor cohort within the high‐level CNV subgroups (CNV 5–10 and ≥ 10). The underlying reason for this association remains unclear and could relate to differences in tumor subtype distribution, hormonal influences, or differential therapy exposure, meriting investigation in future studies with more detailed clinical annotation.

The mutational context of *MET* alterations also differed. The TMB was significantly higher in the lung cancer cohort than in the brain tumor cohort across the overall populations and within *MET* CNV, CNV2‐5, CNV 5–10, CNV ≥ 10 and *MET* segmental amplification subgroups. Only within the *MET* Kinase Domain mutation subgroup, the brain tumor cohort exhibited a significantly higher TMB than the lung cancer cohort. Furthermore, we identified a distinct subgroup of patients harboring *MET* Multi. This cluster was characterized by the highest TMB among all subgroups, suggesting a state of profound genomic instability and intense oncogenic signaling. This profile likely defines a very aggressive, MET‐addicted subset of tumors, which may underlie rapid disease progression and inherent resistance to mono‐therapy. These patients could potentially benefit from more intensive treatment strategies, such as combination targeted therapies or sequential TKI regimens, a hypothesis that warrants prospective clinical investigation.

Our study describes the mutation patterns in a large Chinese cohort. While the primary drivers of *MET* alterations are likely intrinsic genomic instability and selective pressure from therapies, the influence of population‐specific genetic backgrounds or environmental factors cannot be ruled out and warrants further investigation. Comparative studies with matching clinical metadata between Asian and Western populations are needed to dissect the potential contributions of these factors to the observed mutation frequencies.

In lung cancer, *MET* exon 14 skipping and other *MET* alterations generally behave as primary oncogenic drivers, especially in cases where they occur without co‐activating mutations in *KRAS* or *EGFR* [17, 28, 29]. It was observed a high frequency of *TP53* co‐mutation and multiple *MET* alterations in some patients, both of which are associated with genomic instability and may contribute to acquired resistance to MET TKIs or EGFR TKIs [31, 32, 33, 34]. These findings reinforce the need for combination therapy strategies and sequential TKI regimens, particularly for patients with *MET* amplification emerging during EGFR‐TKI treatment. In our study, the rate of *MET* amplification coexisted with *EGFR* mutation was relatively high in lung cancer. *MET* amplification is associated with resistance of EGFR TKIs [35], high histological grades, late clinical stage, and poor prognosis [16, 36]. Several clinical studies have shown that the combination of EGFR TKI and MET inhibitors may be a potential treatment strategy for EGFR TKI resistant patients with secondary *MET* amplification [37, 38].

In the brain tumor cohort, a lower frequency of *MET* exon 14 skipping, but a relatively high incidence of *MET* fusion was indicated. *MET* fusion is rare in clinical practice, but it is very complex and difficult to detect. The previous study has shown 0%–10% of *MET* fusion in brain cancer [23, 24, 39, 40]. Besides the common *PTPRZ1‐MET* fusion, we found several rare or novel *MET* fusions, improving the database of *MET* fusions in clinic detection. In particular, *PTPRZ1‐MET* fusions might be promising therapeutic targets [19, 41]. While these findings are encouraging, it is important to note that the evidence remains preliminary and is primarily based on small cohorts or specific fusion types. Our Simceredx cohort, while identifying several fusions, lacks associated treatment outcome data, highlighting the need for prospective studies to definitively establish the clinical efficacy of MET inhibitors against various *MET* fusions in brain tumors.

The latest NCCN guidelines suggest that patients with *MET* exon 14 skipping may benefit from treatment with crizotinib, or patients with high copy number amplification of *MET* (fold‐change ≥ 10) may benefit from treatment with crizotinib, capmatinib, and tepotinib. However, some patients with *MET* variations were resistant to the MET inhibitor, with the incidence of 4%–19.2% in the treatment of capmatinib, tepotinib, and savolitinib [17, 28, 29]. It is suggested that activating variants in bypass or downstream pathways may be the main mechanism leading to drug resistance. The previous studies have shown that partial drug resistance caused by MET‐dependent mechanism can be resolved by sequential treatment with class I/II MET TKI [8, 42]. However, the clinical evidence is limited; it is necessary to perform clinical trials to verify the safety and efficacy of treatment of MET inhibitors in patients with multiple variants of *MET*.

A real‐world study indicated that the positive proportion of PD‐L1 in NSCLC with *MET* exon 14 skipping was high (84%), but the TMB was low, and the effect of immune monotherapy was poor [13]. In our study, the average of TMB value in lung cancer with *MET* exon 14 skipping was lower than that of the non‐*MET* variation group, while the average of TMB value was relatively high in multiple variants of the *MET* group. For patients with MET inhibitor resistance in NSCLC, the efficacy of immune checkpoint inhibitors alone is limited, and immune combined with chemotherapy and anti‐vascular therapy might be considered [43]. However, the safety and efficacy of ICIs combined with MET inhibitors still need to be further explored.

Targeted therapy has ushered in the era of precision oncology. Significant breakthroughs have been achieved in targeted therapy for *MET* variations. Several limitations of this study should be acknowledged. First, this study includes the reliance solely on DNA‐based NGS testing, without validation through FISH or RNA‐based platforms, which may impose constraints on the accurate detection of *MET* exon 14 skipping and the functional characterization of *MET* fusions. Second, the absence of pre‐clinical and clinical trials where multiple variants of *MET* should be evaluated. Therefore, large‐scale studies are necessary to further assess the safety and efficacy of MET inhibitors or combined therapy in solid tumors with multiple variants of *MET*. Third, for the lung cancer cohort in our Simceredx database, systematic treatment history was not available, limiting our ability to distinguish de novo from acquired alterations in this group. Fourth, our analysis was focused on somatic alterations. Although matched blood DNA was used as a reference to filter germline polymorphisms, a systematic investigation of pathogenic germline mutations associated with hereditary cancer syndrome was not performed. Finally, the absence of detailed clinical metadata, such as smoking history, prevents the analysis of these potential confounders. The prognostic analysis, while supplemented with multivariate analysis for brain tumors using TCGA data, would benefit from validation in independent, prospectively collected cohorts.

In conclusion, we compared the profile and characteristics of different types of *MET* variations in lung cancer and brain cancer and displayed the diverse or conserved mechanisms in different cancer types. Our finding revealed that *MET* is predictive in lung cancer and marks a treatable driver; while in brain tumor *MET* is prognostic and flags a late, focal, high‐CNV program tied to worse survival. This fundamental difference calls for personalized, cancer type‐specific management strategies and future efforts to develop context‐specific interventional strategies targeting MET will be key to achieving more precise cancer therapy.

## Author Contributions

**Yu Zhang:** conceptualization, methodology, investigation, writing – original draft. **Yongmeng Li:** conceptualization, methodology, investigation, writing – review and editing, funding acquisition, resources, supervision. **Dongsheng Chen:** conceptualization, writing – review and editing, resources. **Minghui Ge:** methodology, visualization, supervision. **Ningning Luo:** conceptualization, methodology, investigation, writing – original draft, writing – review and editing. **Yanxiang Zhang:** writing – original draft.

## Funding

This work was supported by the Natural Science Foundation of Shandong Province (grant number ZR2024QH274).

## Ethics Statement

This study was approved by the ethics committee of The First Affiliated Hospital of Shandong First Medical University & Shandong Provincial Qianfoshan Hospital. The requirement for informed consent was waived by the Ethics Committee of The First Affiliated Hospital of Shandong First Medical University & Shandong Provincial Qianfoshan Hospital due to the retrospective and anonymized nature of the data. All procedures were performed in accordance with the Declaration of Helsinki and its later amendments.

## Conflicts of Interest

The authors declare no conflicts of interest.

## Supporting information


**Figure S1:** Comparison of *MET* alteration frequency in recurrence/treated samples and primary samples.

**Figure S2:** Analysis the differences in somatic mutational characteristics between the groups with and without *MET* mutations in the lung cancer cohort (A and B).

**Figure S3:** The differences on TMB in *MET* Kinase Domain (A), *MET* fusion (B), *MET* mutation subgroups (C), *MET* Sema Domain (D) and *MET* multi groups (E) in two cancer cohorts. The differences in TMB between different MET groups in lung cancer cohort (F and G) and in brain tumor cohort (H and I). All comparisons were performed using the Wilcoxon rank‐sum test. A *p* value > 0.05 was considered not statistically significant. Specific *p*‐values are annotated on the figure.

**Figure S4:** The oncoprint plot for the chromosome arm copy number alterations in the brain tumor cohort.

**Figure S5:** The prognosis analysis among the different MET mutation subgroups.

**Figure S6:** The multivariate Cox regression analysis adjusting for age, gender, IDH status, and tumor grade.

**Table S1:** The number of patients in each group.

## Data Availability

The data that support the findings of this study are available on request from the corresponding author. The data are not publicly available due to privacy or ethical restrictions.
