# Supplementary figures and images for: The Mutation Patterns of MET Gene in Lung Cancer and Brain Tumors: Clinical and Therapeutic Implications
Source: Cancer Med. 2026 Jan 12;15(1):e71532. doi: 10.1002/cam4.71532 (PMC12793781; doi:10.1002/cam4.71532)

## Supplementary Figure 1

chisq test p-value<0.001

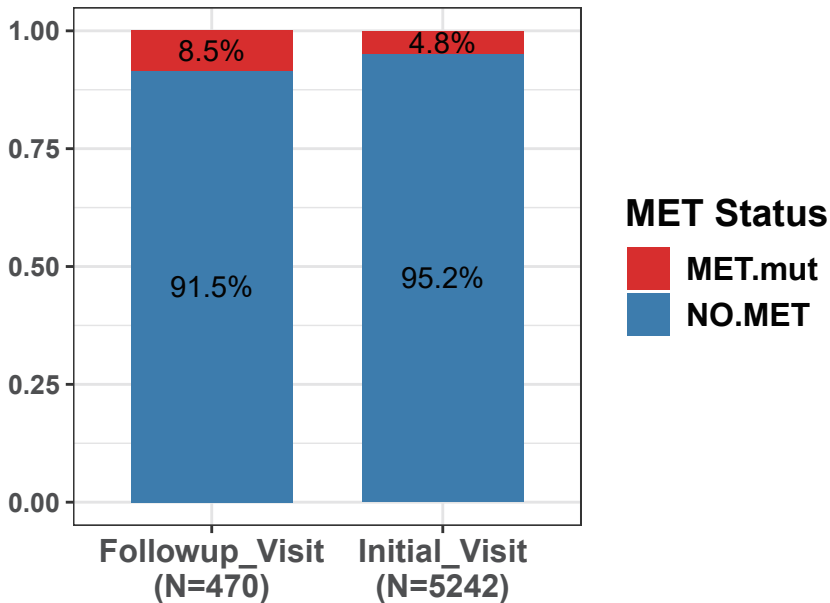

Supplement: Supplementary file 1 — Figure S1: Comparison of MET alteration frequency in recurrence/treated samples and primary samples. [file CAM4-15-e71532-s001.pdf]

Supplementary Figure 2

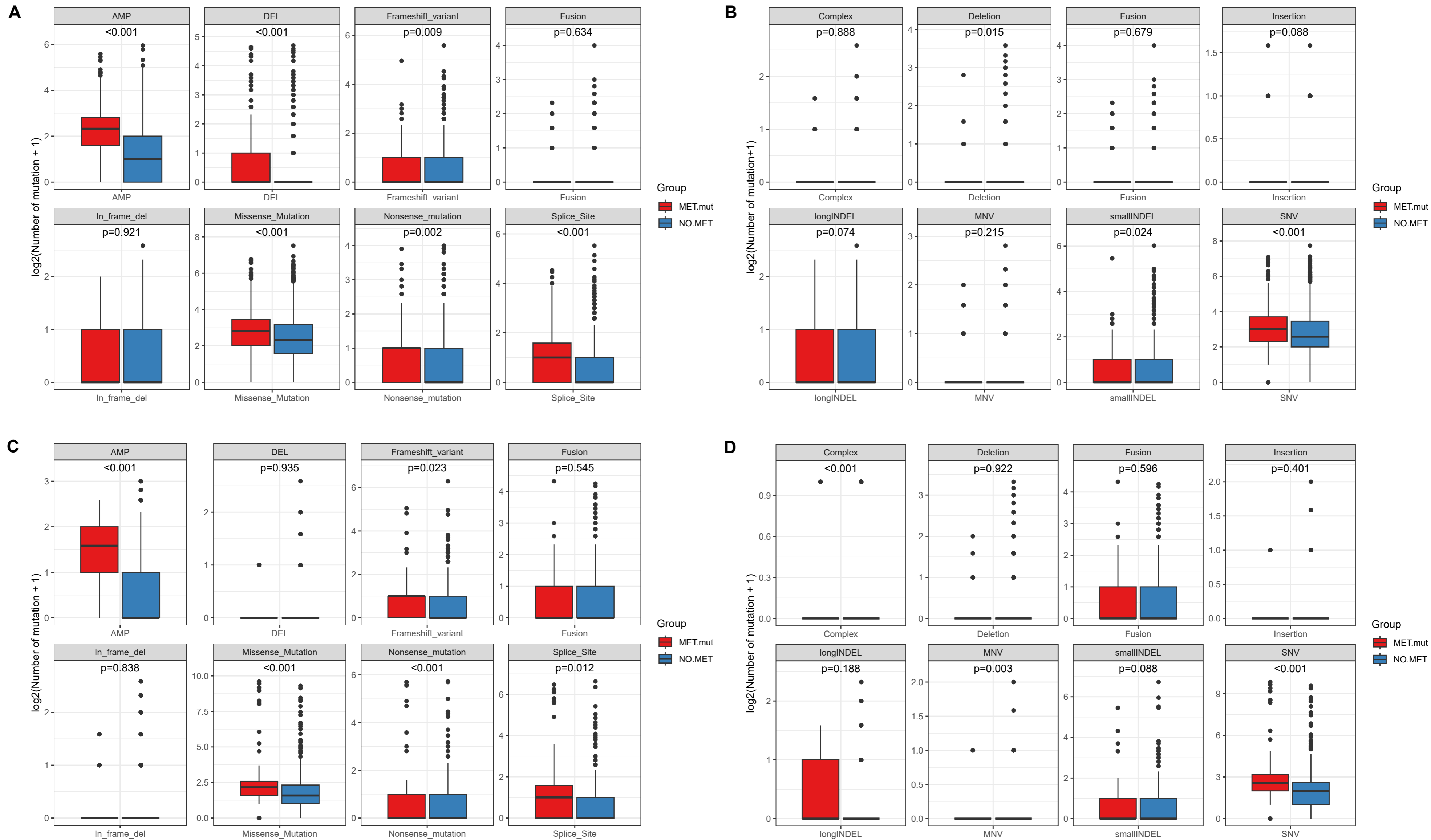

Supplement: Supplementary file 2 — Figure S2: Analysis the differences in somatic mutational characteristics between the groups with and without MET mutations in the lung cancer cohort (A and B). [file CAM4-15-e71532-s002.pdf]

Supplementary Figure 3

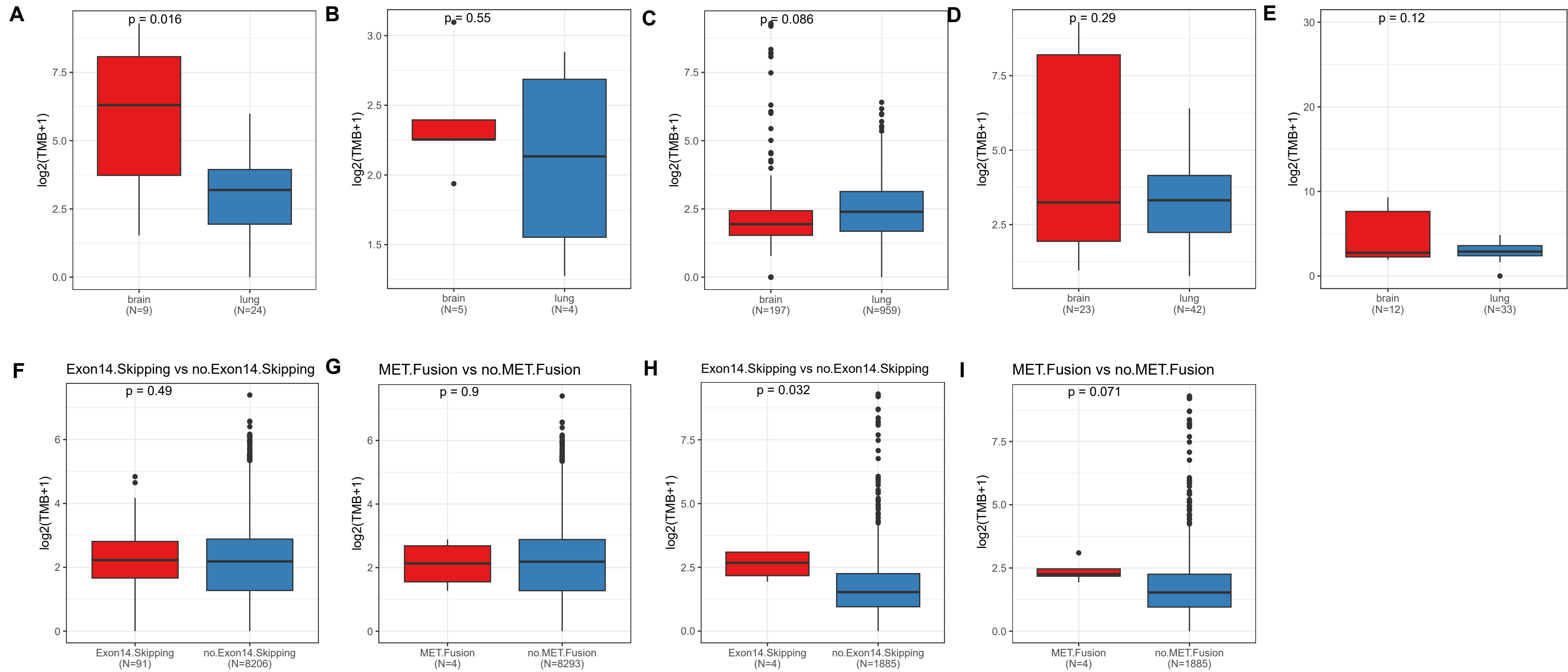

Supplement: Supplementary file 3 — Figure S3: The differences on TMB in MET Kinase Domain (A), MET fusion (B), MET mutation subgroups (C), MET Sema Domain (D) and MET multi groups (E) in two cancer cohorts. The differences in TMB between different MET groups in lung cancer cohort (F and G) and in brain tumor cohort (H and I). All comparisons were performed using the Wilcoxon rank‐sum test. A p value > 0.05 was considered not statistically significant. Specific p‐values are annotated on the figure. [file CAM4-15-e71532-s004.pdf]

Supplementary Figure 4

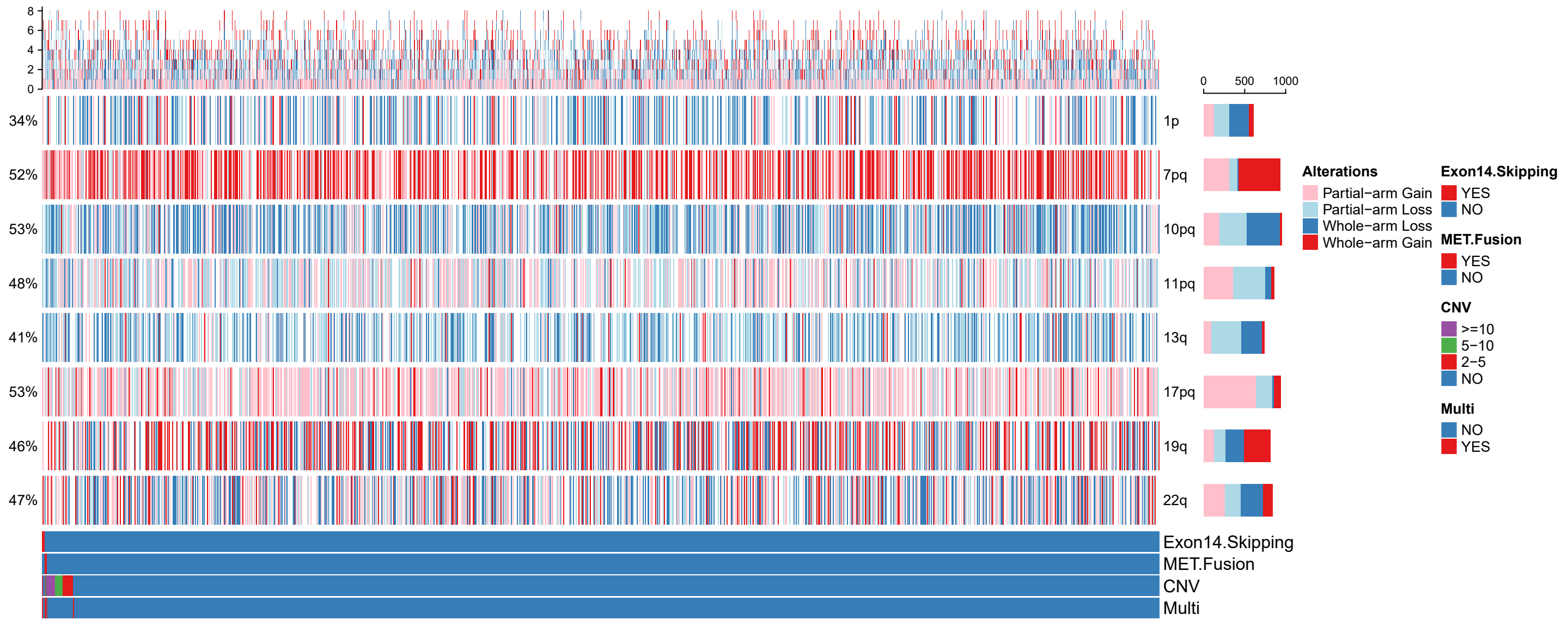

Supplement: Supplementary file 4 — Figure S4: The oncoprint plot for the chromosome arm copy number alterations in the brain tumor cohort. [file CAM4-15-e71532-s003.pdf]

Supplementary Figure 5

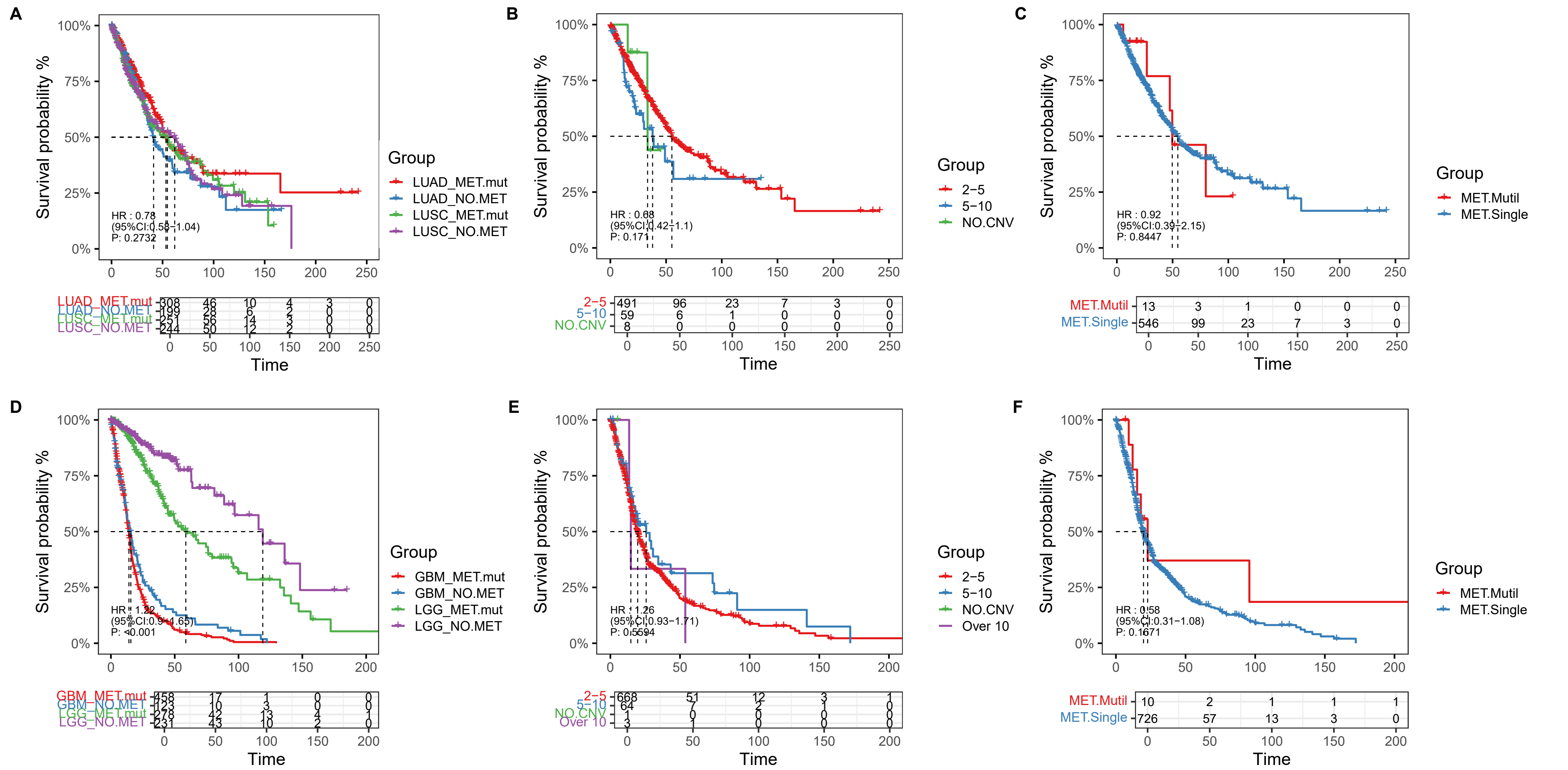

Supplement: Supplementary file 5 — Figure S5: The prognosis analysis among the different MET mutation subgroups. [file CAM4-15-e71532-s006.pdf]

Supplementary Figure 6

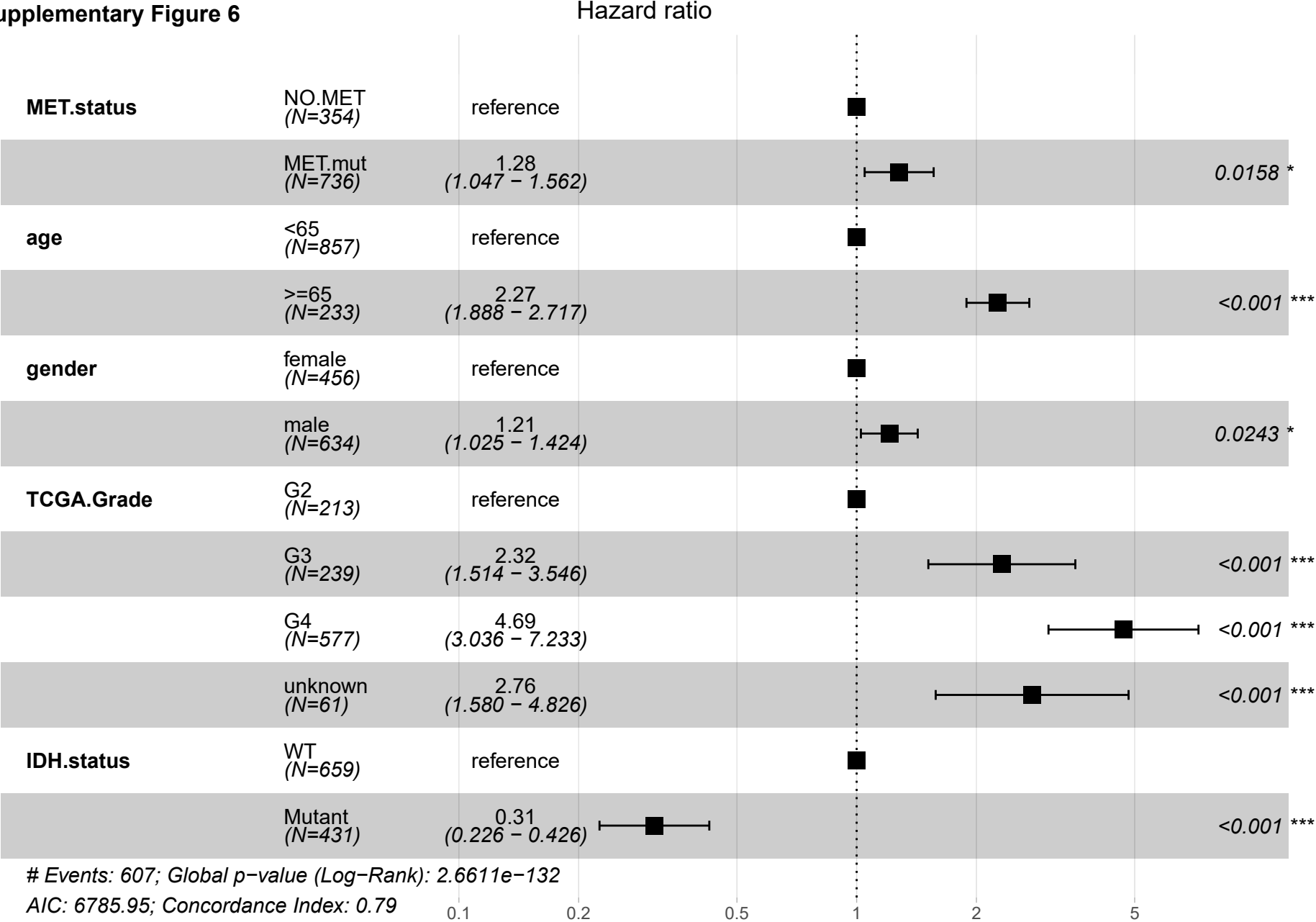

Supplement: Supplementary file 6 — Figure S6: The multivariate Cox regression analysis adjusting for age, gender, IDH status, and tumor grade. [file CAM4-15-e71532-s005.pdf]
