# Supplementary material for: The Mutation Patterns of MET Gene in Lung Cancer and Brain Tumors: Clinical and Therapeutic Implications
Source: Cancer Med. 2026 Jan 12;15(1):e71532. doi: 10.1002/cam4.71532 (PMC12793781; doi:10.1002/cam4.71532)
Supplement: Supplementary file 7 — Table S1: The number of patients in each group. [file CAM4-15-e71532-s007.docx]

**Supplementary Table 1** The number of patients in each group.

| **Simceredx Cohort** | **Lung cancer** | **Brain tumor** | **TCGA Cohort** | **Lung cancer** | **Brain tumor** |
| --- | --- | --- | --- | --- | --- |
| **Total** | 30355 | 6004 | **Total** | 1075 | 1105 |
| **MET var** | 3200 | 331 | **MET var** | 575 | 743 |
| **MET Exon14 Skipping** | 364 | 5 | **MET Exon14 Skipping** | 7 | 2 |
| **MET Fusion** | 15 | 12 | **MET Fusion** | 4 | 3 |
| **MET CNV** | 2407 | 271 | **MET CNV** | 564 | 742 |
| **MET CNV 2-5** | 2159 | 189 | **MET CNV 2-5** | 502 | 675 |
| **MET CNV 5-10** | 188 | 35 | **MET CNV 5-10** | 61 | 64 |
| **MET CNV>=10** | 60 | 47 | **MET CNV>=10** | 1 | 3 |
| **MET Kinase Domain Mutation** | 153 | 23 | **MET Kinase Domain Mutation** | 4 | 5 |
| **MET Sema Domain Mutation** | 398 | 44 | **MET Sema Domain Mutation** | 9 | 3 |
| **MET Muti-mutations** | 131 | 23 | **MET Muti-mutations** | 13 | 10 |
